# Non-coding RNA based regulation of blood vessel development in zebrafish and relevance to humans

**DOI:** 10.1186/1755-8166-7-S1-I10

**Published:** 2014-01-21

**Authors:** Sridhar Sivasubbu

**Affiliations:** 1 CSIR-Institute of Genomics and Integrative Biology, Delhi, India

## 

Non-protein coding RNAs (ncRNA) represent a variety of transcripts having minimal or no protein coding capacity. The ncRNA have been studied with interest for their function as regulators of gene expression. Molecular studies on ncRNA have uncovered diverse interactions with protein coding genes. It has been suggested that ncRNAs are an additional layer of regulatory switches involved in gene regulation during development and disease. Detailed studies deciphering the function of ncRNAs have been limited to a few well-studied candidates. The function of ncRNAs during vascular development has attracted the interest of our laboratory. We designed reverse genetics screens to elucidate the role of miRNAs conserved between human and zebrafish, and potentially involved in regulating vascular development. In this screen we identified several miRNAs involved in the development of vasculature. We also undertook studies to understand transcription factor-miRNA interaction during vascular development. We extended the study to identify long non-coding RNAs (lncRNA) in diverse zebrafish tissues including vascular tissues. We identified several novel lncRNAs from zebrafish tissues. The functional importance of ncRNAs during zebrafish development will be presented and their relevance to humans will be discussed.

